# Graft-Versus-Host Disease: Can Biomarkers Assist in Differential Diagnosis, Prognosis, and Therapeutic Strategy?

**DOI:** 10.3390/ph17030298

**Published:** 2024-02-26

**Authors:** Vaia-Aikaterini Alexoudi, Eleni Gavriilaki, Angeliki Cheva, Ioanna Sakellari, Stavroula Papadopoulou, Konstantinos Paraskevopoulos, Konstantinos Vahtsevanos

**Affiliations:** 1 Oral and Maxilofacial Surgery Department, G Papanikolaou Hospital of Thessaloniki, 57010 Thessaloniki, Greece; valexoudi@yahoo.gr (V.-A.A.); kostparas@yahoo.gr (K.P.); vaxtseva@gmail.com (K.V.); 2 2nd Propedeutic Department of Internal Medicine, Aristotle University of Thessaloniki, 54124 Thessaloniki, Greece; 3 Department of Pathology, School of Medicine, Aristotle University of Thessaloniki, 54124 Thessaloniki, Greece; antacheva@yahoo.gr; 4 BMT Unit, Hematology Department, G Papanikolaou Hospital of Thessaloniki, 57010 Thessaloniki, Greece; ioannamerilena@gmail.com; 5 Department of Pathology, G Papanikolaou Hospital of Thessaloniki, 57010 Thessaloniki, Greece; staupapad@gmail.com

**Keywords:** biomarkers, GVHD, saliva

## Abstract

A crucial complication after allogeneic hematopoietic cell transplantation (alloHCT), namely, acute graft-versus-host disease (aGVHD), occurs in about 50% of transplant recipients, leading to high morbidity and mortality. Thus far, the diagnosis of GVHD has been mainly established through clinical features and histologic or laboratory evidence of periductal lymphocyte infiltration, fibroplasia, and mixed lymphocytic and plasmocytic inflammation. Intensive research is focused on identifying biomarkers for the early diagnosis, prediction of disease, response to treatment, prognosis, and risk stratification of patients. The serum biomolecules that have been investigated are reported and summarized. Moreover, oral tissue involvement in GVHD is described, and other biomarkers that have been proposed, such as saliva, are analyzed. Future research is highlighted as a necessity in order for these biomarkers to be validated and quantified for use in clinical practice.

## 1. Introduction

AlloHCT is one of the most effective and frequently performed treatment strategies used for conditions such as hematological malignancies, hemoglobinopathies, and others [1]. The higher life expectancy ratios also indicate higher risks for post-transplantation complications, including graft-versus-host disease [2].

A crucial complication after alloHCT, namely, aGVHD, occurs in about 50% of transplant recipients leading to high morbidity and mortality [3]. GVHD is initiated by the recipients’ antigen-presenting cells (APCs), termed alloantigen presentation. Once recipients’ APCs are eliminated post-transplantation, donor-derived APCs sustain GVHD by presenting recipient-derived peptides through an indirect pathway. aGVHD is characterized by severe damage to the gastrointestinal (GI) tract, liver, skin, and other mucosal tissues [4]. Generally, patients with aGVHD are treated by optimizing immunosuppression and adding corticosteroids. The GI tract manifestations of aGVHD are the most severe and resistant to treatment and pose substantial diagnostic and therapeutic challenges [5,6]. Therefore, the GI tract is considered to play a pivotal role in the propagation of the disease and is a focus of research interest. Oral manifestations in aGVHD are rare and nonspecific. These may include erythema, with the presence or absence of ulcers on the oral mucosa or lips [7].

In addition, chronic graft-versus-host disease (cGVHD) is a major cause of morbidity and mortality in alloHCT patients. cGVHD constitutes an immune-mediated complication in alloHCT patients that can affect various organs of the body, with oral features presenting among affected patients at rates of 45% to 83% [1].

The current diagnoses of aGVHD and cGVHD rely on clinical information, supplemented with evidence from pathological findings in the affected tissues. However, relying on clinical suspicions and waiting for pathology results may lead to therapy delays and suboptimal clinical outcomes. Obtaining biopsies, especially for aGVHD, can be challenging due to the risk of bleeding in hepatic samples. In contrast, oral samples in cGVHD offer easier access, facilitating early syndrome diagnosis.

Unfortunately, there are no up-to-date approved biomolecules for the early detection of both syndromes. Having such biomolecules would be beneficial, saving time and aiding in identifying patients who require early therapy.

This article aims to summarize ongoing research on biomolecules for diagnosing, predicting, and assessing the risk of GVHD. It also reviews the oral perspective of the syndrome as a potentially advantageous environment for detecting the condition.

## 2. Protein Biomarkers for Acute and Chronic GVHD

A biomarker is a measurable and verifiable characteristic that can be implicated in a biological, pathogenic, or pharmacological response process [8]. In the case of GVHD, both acute and chronic, it is of major importance that biomarkers be traced within an optimal timeframe so that treatment can commence promptly. The proposed biomarkers can be categorized into proteomic (soluble or cellular, implicating inflammatory cells), as well as genetic (microRNAs—miRNAs) or small nuclear particles (SNPs).

**Figure 1:**
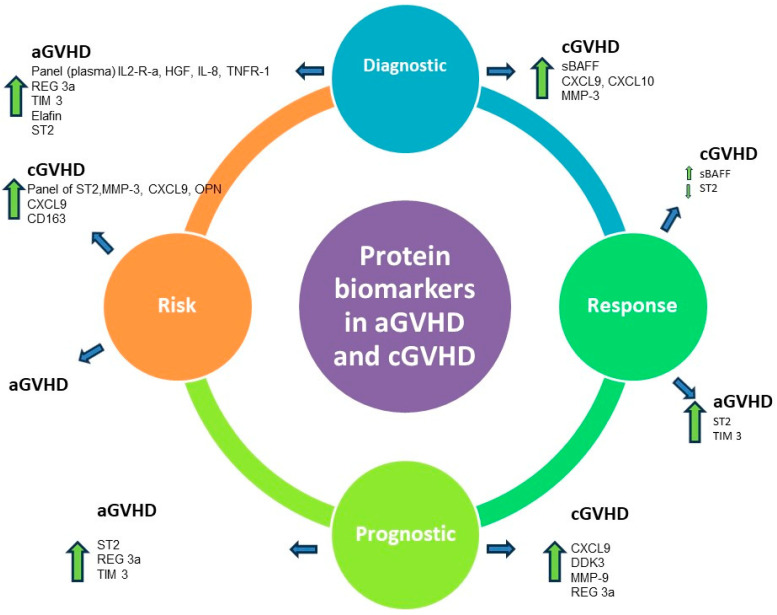
IL2-R-a: Interleukin 2 Receptor a; ST2: Suppressor of Tumorigenicity 2; HGF: Hepatocyte Growth Factor; TNFR-1: Tumor Necrosis Factor Receptor-1; Reg-3a: Regenerating Islet-Derived Protein 3a, a peptide found in Paneth cells of the intestines (the most validated GI aGVHD biomarker); TIM 3: T-cell Immunoglobulin Mucin Protein 3; Elafin: a serine protease inhibitor; sBAFF: Soluble B-Cell Activating Factor; CXCL9: Chemokine (C-X-C motif) Ligand 9; CXCL10: Chemokine (C-X-C motif) Ligand 10; DDK3: Dickkopf-Related Protein; MMP-3: Matrix Metalloproteinase 3; MMP-9: Matrix Metalloproteinase; OPN: Osteopontin; CD163: a scavenger receptor by activated monocytes/macrophages found in cases of oxidative stress. 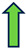: Increased levels; 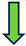: decreased levels.

In addition, according to the NIH BEST Resource, biomarkers applicable in GVHD can be subcategorized as diagnostic, predictive, response, prognostic, and risk. The recommended practices for biomarker development include discovery, validation, and qualification research, which highlight the difficulty in establishing a marker for clinical practice [9]. As far as GVHD is concerned, the protein biomarkers that have been examined can be summarized and divided into aGVHD and cGVHD potential biomarkers (Table 1 and Figure 1).

The pathophysiology of aGVHD is suggested to be a three-phase phenomenon involving: 1. inflammatory damage to host epithelial tissues subsequent to the preparatory chemotherapy and radiotherapy regimen (i.e., conditioning); 2. recipient and donor APCs and inflammatory responses inducing the activation of donor-derived T cells which, in turn, expand and differentiate into effector cells; and 3. cytotoxicity mediation by activated donor-derived T cells against host cells through mechanisms that involve proinflammatory cytokines, such as tumor necrosis factor (TNF) a and interleukin-1 (IL-1), IL-6, IL-10, and IL-12 [7]. Despite the progress in the field, the mechanisms and effector molecules behind the manifestation of aGVHD are not fully understood. 

As shown in Table 1, various plasma and organ-specific biomolecules have been examined to assess their potential utility as biomarkers, with a predominant focus on aGVHD and the significant ongoing efforts in the field of cGVHD research. However, it is emphasized that further research, particularly in the form of cohort studies, is still required.

A panel of plasma markers, including IL-2Ra, HGF, HL 8, and TNF R1, was tested as a diagnostic biomarker, in 2009, in a cohort of 424 patients, helping to identify aGVHD at the onset of symptoms [10]. 

As previously mentioned, apart from plasma biomarkers, organ-specific biomarkers have been tested, for aGVHD. These include regenerating islet-derived protein 3α (REG 3a), a peptide that increases in Paneth cells at the onset of aGVHD. Furthermore, T-cell immunoglobulin mucin protein 3 (TIM 3), a serine protease inhibitor (Elafin), and suppressor of tumorigenicity 2 (ST2) have been proposed as organ-specific biomarkers [15,17,19,20]. 

ST2 and TIM 3 have been investigated as potential response biomarkers in the case of aGVHD by Mc Donald et al. 2017 [21]. A validated response biomarker would be beneficial in evaluating the effectiveness of novel therapies.

Reg 3a and ST2 have been proposed as potential prognostic biomarkers in several studies, with increased levels associated with nonrelapse mortality (NRM) [15,20]. Additionally, efforts have been made to develop an algorithm (MAGIC) that combines biomarkers to serve as a prognostic marker [28,29]. ST2 and REG3a are considered two of the MAGIC biomarkers, aiding in the identification of steroid-resistant patients (Mayor-Monfried). However, none of the conducted studies have reached the point of validating these biomarkers for use in clinical practice. The same applies to risk biomarkers for aGVHD [9].

So far, among the protein biomolecules that are elevated in GHVD, those with the most research and greatest relevance to aGVHD are ST2 and regenerating islet-derived protein 3α (Reg3α) [30].

ST2 is an alias for interleukin-1 receptor-like-1 (IL1RL1), defined as the receptor of IL-33, according to the Human Gene Nomenclature Database. The ST2 gene encodes for two products resulting from alternative splicing and 3’ processing at the RNA level [6]. They include a membrane receptor of the IL-1 receptor family, named membrane ST2 (mST2), and a truncated soluble ST2 (sST2) form of the receptor that can be detected in human serum. Both forms of the receptor contain the same binding site for IL-33. 

IL-33 is a nuclear protein that belongs to the IL-1 family of cytokines that is found in lymphoid organs, which is also constitutively expressed in endothelial and epithelial barrier surfaces. IL-33 is also known as an “alarmin” or an endogenous danger signal [5,31]. IL-33 is rapidly increased in response to epithelial tissue injury and signals via the mST2 receptor to initiate and amplify inflammatory responses through the activation of the NFkB and MAP kinase signaling pathways [31]. The soluble form of ST2 is proposed to act as a decoy receptor, sequestering IL-33 and limiting its activity [6,31]. 

Both circulating ST2 and/or IL-33 have been suggested to be elevated in certain pathological conditions, including myocardial infarction, asthma, urticaria, heart failure, and, most recently, in resistant aGVHD [5,20,31,32,33,34,35]. Conversely, high levels of serum sST2 are a marker of poor prognosis in cardiovascular disease. Many recent findings strongly suggest a functional role of the IL33/ST2 axis in the modulation of aGVHD in preclinical studies, while high levels of sST2 and IL-33 in the serum have been suggested as a marker for the risk of therapy-resistant GVHD and death [31,33]. The latter may appear to be contradictory considering that sST2 in mice models acts as a scavenger for IL-33 preventing it from exerting its proinflammatory role. An explanation might be that the high sST2 levels detected in patients may be due to the reaction of the immune system to counteract IL-33-mediated inflammation [36]. In addition, other mechanisms exist to control IL-33 bioactivity including mechanism of release, N-terminal processing, oxidation, and regulation of mST2 expression [31].

Despite the fact that IL-33/ST2 signaling has been well documented as a crucial mechanism in the onset and severity of aGVHD, particularly manifested in the GI tract of both murine models and stage IV human patients, the collected data seem to reveal a paradoxical role of IL-33 in the development of the disease [20,33,34,37]. It seems likely that IL-33 exerts different immune responses depending on the disease model, cell type, and inflammatory milieu [36]. As a matter of fact, IL-33 may have pro- or anti-inflammatory effects in aGHVD, and this phenomenon is regulated spatially and over time [33]. It has been shown that the absence of IL-33 in experimental models results in decreased GVHD severity through a mechanism that involves the suppression of TNFa release, and this phenomenon is inverted when IL-33 is administered followed by an increase in the number of effector T cells. Additionally, using sST2 to block IL-33 from binding to mST2 on donor T cells reduces inflammatory cytokines, attenuates GVHD damage to the GI tract, and improves survival. The paradox of IL-33 is revealed when IL-33 is administered to murine models prior to alloHCT. It appears that early administration of IL-33 before transplantation has a “protective” effect, since it results in the prevention of macrophage activation and a decrease in the number of effector T cells that damage GVHD target tissues [33,34]. 

To summarize, the mechanism regulating aGVHD through IL-33/ST2 signaling is complex and remains to be fully elucidated. According to a well-established recent model, a simplified sequence of events may be as follows: The conditioning regimen performed in alloHCT causes damage to the GI epithelium and underlying fibroblastic reticular cells inducing them to produce IL-33 [33,34]. IL-33 then activates donor Th1 cells by binding to mST2; subsequently, Th1 cells differentiate and expand, promoting GVHD and further epithelial damage [34]. The sST2 serum concentration is suggested to be a predictor of GVHD severity and steroid-resistant aGVHD, and it most likely reflects the activation of and damage to the GI mesenchyme. 

Reg 3a, mentioned earlier in this section, is likely an indicator of damage to and activation of the GI epithelial compartment and should be evaluated in combination with sST2 for the prediction of the long-term outcomes of GVHD. Reg-3α exhibits antibacterial effects against Gram-positive bacteria and is regarded as a byproduct during the breakdown and regeneration of intestinal tissue. Studies have indicated that the concentration of Reg-3α on days 7 and 14 post-HSCT serves as a reliable predictor for acute GVHD and nonrelapse mortality (NRM) [38,39].

Elafin is alternatively known as a skin-derived antileucoprotease and is present during the repair process of skin lesions [19,40].

Other protein biomarkers identified in serum for aGVHD detection include plasma amphiregulin/EGF ratio, plasma IL2Ra, TNFR1, soluble B-cell activation factor (BAFF), chemokine (C-X-C motif) ligand 10 (CXCL10), chemokine (C-X-C motif) ligand 11 (CXCL11), and serum albumin [41].

In the case of cGVHD, the utility of BAFF and its ratio with B cells has been assumed to be one of the possible biomarkers, although its diagnostic utility has been questioned due to the fact that it is influenced by the corticosteroid therapy that HCT patients receive [11,12,13,14,37].

Other biomarkers being researched are the inflammatory chemokines CXCL9 and CXCL10, which were found to be increased in the case of a diagnosis of cGVHD in some studies. Furthermore, a panel of ST2, CXCL9, and matrix metalloproteinase-3 (MMP-3) has been found to correlate with the diagnosis of cGVHD. Other biomarkers that may indicate cGVHD and its possible early diagnosis are Dickkopf-related protein (DDK) and REG 3a, which have been investigated in aGVHD and cGVHD [13,14,16,24,25].

There is a need for further investigation of diagnostic biomarkers for cGVHD due to the fact that the early signs and symptoms may not be specific, and patients may only be diagnosed at an advanced stage of the condition.

Moreover, there are no currently validated predictive biomarkers for cGVHD [32].

Other biomolecules that are under research for the prognosis of cGVHD include CXCL9, DKK3, MMP-9, and REG 3a. Increased levels have been detected in each case, even though more studies are required in order for these molecules to be used in clinical practice [23,25,27].

To monitor the treatment response, studies have been conducted with BAFF and ST2, yet the validation and quantification of these biomolecules have not been achieved [22].

Finally, in the field of risk biomarker research for cGVHD, there seems to be promising results using a four biomarker panel in order to predict future disease. The panel consists of ST2, CXCL9, MMP-3, and OPN at 100 days post-HCT. CXCL9 and CD 163 levels have also been investigated as potential risk biomarkers for these patients. It would be beneficial in clinical practice to know in which patients’ immunosuppression could be tapered and in which patients this would not be an option [27,37].

## 3. GVHD and Transplant-Associated Thrombotic Microangiopathy (TA-TMA)

In the last four years, there has been intensive research that associates GVHD, acute or chronic, with TA-TMA. Both conditions share common characteristics and have endothelial damage as a common denominator. More specifically, Gavriilaki et al. conducted a survey enrolling HCT recipient patients with TA-TMA, aGVHD, or cGVHD, as well as controls. They demonstrated that complement activation is present in endothelial damage syndromes, specifically higher in patients with TA-TMA. Moreover, an index called EASIX, taking into consideration platelet count, LDH, and creatinine, can be used as a prognostic biomarker in the population of both GVHD and TA-TMA patients, reflecting the underlying endothelial damage [42].

Endothelial injury in patients with acute or chronic GVHD using circulating microvesicles has also been investigated showing increased thrombotic risk and endothelial dysfunction in these patients [43].

Furthermore, TA-TMA has been associated with genetic susceptibility in HCT patients, which was not evident in the controls, according to the research of Gavriilaki et al. [44]. Therefore, post-transplant endothelial damage may be a future genomic research field.

## 4. Genetic Biomarkers for aGVHD and cGVHD

MicroRNAs (miRNAs) are short noncoding RNAs, typically around 20–25 nucleotides in size. They operate by binding to messenger RNAs (mRNAs) and primarily control gene expressions [8]. In recent years, there has been a rapid evolution in the study of miRNAs, particularly in the field of cancer, shedding light on numerous particles that hold potential as biomarkers.

Similarly, during the last decade, interest has escalated in GVHD research, which may be due to the fact that although proteins as biomarkers may seem to be more informative than miRNAs, the latter have been reported to be more stable in body fluids, and they may be measured using quantitative PCR. Moreover, the technological advancements in bioinformatics have made possible the registration and processing of the available genetic data [45].

Regarding allografting, the majority of research has focused on aGVHD and the potential involvement of T cells in the syndrome’s pathophysiology. Notably, miR-155 has emerged as one of the initially proposed genetic biomarkers, exhibiting upregulation in cases of aGVHD. Moreover, the upregulation of miR-181a in rodent models appears to play a potential preventive role in the onset of aGVHD. Clinical trials indicate a reduction in miR-181a expression before the onset of aGVHD [46,47].

Recently, research has highlighted further potential genetic biomarkers for aGVHD, although none of them have been validated for use in clinical practice [38]. A study by Ward Pa et al. proposed that the expression of a negative regulator of inflammation, such as miR146a, may serve as protection against aGVHD onset [48].

Other particles that seem to be upregulated in the case of aGVHD include miR20a and -15a. In contrast, the downregulation of other particles, such as miR146a, miR30b-5p, and miR374-5p, has been reported in patients with aGVHD [49]. In addition, specific particles, such as miR34a-3p and miR503-5p, seem to overexpress on samples from target organs such as skin [50]. It is suggested that miRNA clusters may be more informative than single miRNAs [38]. Other research proposes the use of a panel based on four miRNAs after the observation that the above may predict the occurrence and severity of aGVHD [51].

Concerning cGVHD, both single- and miRNAs have been found to play roles as biomarkers. More specifically, Reikvam et al. developed a potential diagnostic biomarker model for cGVHD through miRNA sequencing analysis of serum samples of allo HCT patients. Further and larger studies are needed in order to validate these findings [52]. Moreover, miR-17-92 has also been implicated in the pathogenesis of cGVHD in a study that demonstrated that miR-17-92 has an important role in the development of scleroderma in cGVHD patients [53] (Table 2).

## 5. SNPs as Biomarkers in GVHD

It has been established that most genetic variations among humans consist of single nucleotide polymorphisms (SNPs) and, thus, different proteomic products. SNPs have been identified as possible biomarkers for aGVHD and cGVHD. The most studied particles include TNF-a, IL-6, interferon [IFN]-c, IL-10, and UDP-glucuronosyltransferase 2B17) [54,55]. On the basis of the above, non-HLA polymorphisms may result in acute or chronic GVHD. However, SNP-genotyping-based donor selection has not been carried out. The need for research in the genetic field is obvious in order to identify more genetic polymorphisms [8]. 

An IL-23 receptor polymorphism has been associated with a reduction in the occurrence of GVHD, and this polymorphism is also linked to a decrease in the function of CD4 and CD8 T cells [56,57,58]. Additionally, there is documentation of a protective function of the IL-23/IL-22 pathway in an experimental model of intestinal GVHD [59].

Furthermore, recently, Mougeot et al. proposed an SNP associated with cGVHD. More specifically, they used saliva samples from HCT patients and performed exome sequencing, finding three genes in chromosome 9, which may show susceptibility to cGVHD. More research in this field may highlight further genetic polymorphisms concerning cGVHD [60].

Genetic studies seem to be promising, considering the explosive data collection through bioinformatics; however, in order for biomarkers to be used in clinical practice, more studies are required in this field.

## 6. Oral Biomarkers for GVHD

Currently, the oral environment is attracting attention as a novel area of interest. Biopsies are conducted to achieve an early diagnosis of GVHD, especially chronic GVHD. 

The aforementioned is significant due to the absence of biomarkers currently accessible in clinical settings.

It is widely recognized that systemic diseases can affect the oral environment, and practitioners often overlook this easily accessible and diagnostic area. Numerous studies, as provided below, have investigated the clinical manifestations of various conditions, including GVHD.

Haverman et al. emphasized the importance of allo-HCT patients consulting oral health practitioners for early identification of oral conditions arising from their medical status. In their research, the authors investigated oral outcomes following cellular therapy aimed at treating diverse hematological diseases, both malignant and nonmalignant [1]. They observed an increased likelihood of developing oral features, such as superficial mucoceles, trismus, lichenoid lesions, and heightened sensitivity of certain mouth regions to various foods [61]. These manifestations occur post-transplantation of donor stem cells or bone marrow into the recipient’s body, leading to specific clinical conditions. The most affected targeted tissues are the nonkeratinized tissues, which mostly involve the oral buccal mucosa and the perioral tissues [62]. Mucosal sensitivity, pain, dry mouth, and taste alterations have also been reported. All of the above conditions may have an immediate effect on the everyday life of patients by compromising daily function and nutrition and contributing to limitations in oral health care. In addition, oral candidiasis, infections, and caries are possible, as well as secondary oral malignancies [63,64]. The present oral challenges make it necessary to optimize oral hygiene with regular check-ups and diagnosis after such procedures.

An up-to-date diagnosis of cGVHD, according to the National Institutes of Health (NIH) criteria, is established mainly by clinical features and histologic or laboratory evidence of periductal lymphocyte infiltration, fibroplasia, and mixed lymphocytic and plasmacytic inflammation. Most of the time, the biopsy location for proper diagnosis is acquired from the labial minor salivary glands, which contain the aforementioned pathological features.

So far, the proposed model of immunoreconstruction includes normal immune restoration of protective immunity with host tolerance, developed functional tolerance with graft-versus-tumor effects, as well as immune system alloreactivity that may lead to aGVHD and cascade to cGVHD [65]. 

cGVHD, as a multiorgan autoimmune disorder with major risks, influences the everyday quality of life of these patients. This encompasses the oral setting, which significantly influences quality of life by playing crucial roles in the functions such as chewing, swallowing, speech, and facial appearance. Therefore, the need for specific biomarkers in everyday clinical practice is obvious and will allow for better prognosis and treatment protocols for these patients [41].

Crossland et al. reviewed the models generated in identifying novel biomarkers for disease development. The proposed biomarker research, according to Crossland et al., encompasses alloantibodies, glycomics, endothelial-derived particles, extracellular vesicles, and microRNA and DNA methylation products. Nonetheless, further research, validation and correlation between clinical and laboratory findings are needed for the biomarkers to be used in clinical practice [66].

Another comparison is derived from the ideas presented by Zhang et al., who attempted to define the occurrence of novel biomarkers in patients with oral squamous cell carcinoma (OSCC). The study evaluated the presence of the biomarkers for the diagnosis and possible treatment direction in patients with OSCC [67].

Additionally, another study suggested that the application of targeted treatments based on biomarker research may lead to a reduction in GVHD in hematologic patients, therefore enhancing their quality of life [41].

Moreover, other tissues apart from serum, such as saliva, have been evaluated as convenient and noninvasive in identifying the presentation of aGVHD by Zhao et al., who report that saliva consists of organic and inorganic solutes, as well as peptides, which relate to the immunity of the host. Presumably, changes in the amount of the aforementioned may be investigated in people who develop autoimmune diseases such as graft-versus-host disease [68].

In addition, Chiusolo et al. identified two saliva proteins as potential biomarkers of aGVHD and mucositis in the specimens of patients who had received allo-HSCT using liquid chromatography combined with electrospray–ionization mass spectrometry [69].

In addition, the concept of biomarkers in saliva specimens has been also used in the proteomic study of Costa-da-silva et al., in which whole saliva specimens were used in order to assess the protein secretion changes in patients with c-GVHD. In conclusion, ZG16B, a secretory lectin protein, was found to be reduced twofold in patients with the syndrome, indicating general salivary dysfunction [70]. Certainly, further investigation is required in this domain.

Studies have highlighted that the potential use of biomarkers in the assessment of a disease is in line with clinical prognosis, diagnosis, or prediction [66,68]. Oral samples were examined for changes in molecular and cellular biomarkers, exploring their potential applications in diagnosis and treatment. The diagnostic efficacy of oral biomarkers lies in their capacity to be identified and linked to the onset of a specific condition, aiding in accurate identification. According to Zhao and Huang, effectively managing cGVHD relies on early diagnosis and intervention. Their research demonstrated that recognizing a specific biomarker can facilitate the prediction, prognosis, and diagnosis of the condition. The overarching concept of a potential biomarker has been linked to risk stratification, enabling clinicians to anticipate the likelihood of graft development leading to malignancy or other associated conditions [68].

A study by Lorenzo-Pouso et al. found the presence of several biomarkers in the mouth to be associated with certain ailments [71]. The study focused on specific oral conditions and associating them with certain biomarkers. The authors concluded that saliva can be used as a base tool for the identification of biomarkers in the prediction, prognosis, diagnosis, and treatment of a plethora of conditions. cGVHD can be included in the above conditions influencing the oral tissues and predisposing the organs to the development of a disease.

## 7. Potential Role of Biomarkers in Disease Detection

Research has identified OSCC as one of the most frequent cancers worldwide. A study by Riccardi et al. investigated the possible use of salivary biomarkers in the diagnosis of OSCC. Although the gold standard for the diagnosis is biopsy, the study suggests using technology to recognize the presence of specific biomarkers in the diagnosis of OSCC. The oral biomarkers were associated with the efficient determination of the presence of such conditions as OSCC, where more than a hundred possible saliva biomarkers were identified [4].

A different study by Maheswari et al. attempted to predict the possibility of oral mucosal disorders becoming malignant through biomarkers. The researchers reported a significant percentage, between 16% and 62%, of epithelial dysplasia, which may lead to malignant transformation. The critical findings of the review were based on the use of micro-RNA to detect the occurrence of abnormalities in patients with oral mucosal disorders. The interpretation of the development of abnormalities results from the functionality of the potential biomarker, micro-RNA, which is highly associated with the cell regeneration and development of oral tissues. The associated functionality is related to the need for deregulation, which is among the most detectable signatures of oral carcinogenesis [72].

In addition, the potential use of biomarkers in aGVHD has been investigated over the past decade. An article published in 2019 provides relevant insight regarding the potential of the new novel biomarkers for aGVHD. Srinagesh et al. generated insight regarding the presence of serum biomarkers in the body of the host and the transplantation graft. Specifically, the discovery and validation of GVHD biomarkers is an objective of 25 hematopoietic cell transplantation centers. Such information is relevant to predictive prognosis and diagnosis. The disease risk of each patient can be estimated, monitored, and strategized in order to adopt the best treatment response [73].

Another cohort study used the ocular surface as the base evaluation for the presence of biomarkers. The baseline was evaluated for specific markers’ recognition for cGVHD. The assessment and classification of the baseline resulted in tendencies of ocular abnormalities. The specific characteristics, such as ocular dryness, were related to hematological disorders, showing the possibility of the development of the condition. The results may have an impact and may be used as oral biomarkers. The findings are supported by another article that suggests an in-depth analysis of oral biomarkers in tracking the response of cells to treatment therapy for cGVHD [74]. In their study, Rozmus and Schultz [75,76] focused on the biological origination and assessment of the biomarkers toward structuring a more comprehensive framework to recognize the development of GVHD. The research highlighted present challenges and upcoming paths in the GVHD domain, aiming to identify the most suitable treatment options. The researchers suggest that novel biomarkers could play a crucial role in evaluating cGVHD, extending beyond serum samples to include saliva and urine. Overall, biofluids, including saliva, tears, and urine, have been reported as appropriate samples for the identification of potential biomarkers for disease detection.

All of the above is in line with the idea that biomarker discovery has emphasized the need to find protein markers before the symptoms of a disease appear so that a proper stratification of a patient as high or low risk may be possible in order to follow the proper treatment [41].

As far as cGVHD is concerned, the need for further proteomic and transcriptomic studies is highlighted. This may be among other reasons because biomarkers levels are highly influenced by drugs used in immunosuppression, such as corticosteroids. It is obvious that biomarker research should focus on the tissues affected by the syndrome, including the skin or the mouth [41].

Biomarker use has been suggested to be important in the detection of several diseases. In a study conducted by Rezasoltani et al., the effectiveness of novel biomarkers for colorectal cancer screening was examined [76]. The study investigated the presence and prevalence of a biomarker, microbiomes, in both oral (i.e., saliva) and fecal samples. A comparison of the cluster density revealed alpha-domination in the oral samples and beta-domination in the fecal samples in terms of the population of the novel biomarker. The study’s findings suggest that saliva could be utilized for more effective detection of conditions like colorectal cancer. These results align with a recent systematic review by Pillai et al. that focused on proteomic biomarkers in the analysis of OSCC [77]. The conclusions of the study termed the biomarker as a protein signature whose expression can be a sign of the development of abnormalities in cell regeneration and growth. With the use of a biomarker, clinicians can determine the development of the disease through prediction, diagnosis, and structured individualized therapy in the treatment process.

## 8. Conclusions

The exploration of biomarkers has become increasingly popular in recent years, aiming to aid in diagnosing, predicting disease outcomes, and assessing treatment responses, as well as evaluating the risk of disorders like GVHD. Novel biomarkers can be identified through the analysis of various biofluids, such as serum, saliva, and urine.

Regarding aGVHD, numerous potential biomarkers have been identified in research, with many studies emphasizing, among others, the combined use of ST2 and REG-3a in the MAGIC algorithm as a valuable tool for early syndrome detection. However, for cGVHD, promising biomarkers have not yet been clearly identified, although ongoing efforts are actively evaluating potential candidates.

The role of biomarkers in triggering alloreactivity leading to GVHD has been established through their evaluation. Their practical application in clinical settings is evident, particularly in the early detection and treatment of these conditions. Despite ongoing research, challenges persist in the discovery, validation, and quantification of biomolecules, making their current utilization challenging. Prospective cohort studies are crucial for the proper validation and quantification of potential biomarkers, considering that, beyond serum, other biofluids may also prove beneficial.

## Figures and Tables

**Table 1:** Protein biomarkers research for aGVHD and cGVHD.

-	aGVHD	cGVHD
-	Protein Biomarkers	
Diagnostic	Panel (plasma) IL2-R-a, HGF, IL-8, TNFR-1 [10] 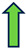	sBAFF [11,12,13,14] 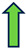
-	Reg-3a [15] 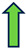	CXCL9, CXCL10 [14,16] 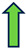
-	TIM 3 [17] 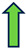	MMP-3 [18] 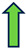
-	Elafin [19] 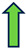	-
-	ST2 [20] 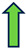	-
Response	ST2 [21] 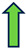	sBAFF [22] 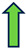
-	TIM3 [21] 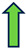	ST2 [9] 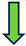
Prognostic	ST2 [20] 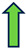	CXCL9 [23] 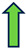
-	REG3a [15] 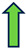	DDK3 [24] 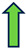
-	TIM3 [9] 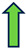	MMP-9 [25] 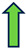
-	-	Reg-3a [26] 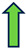
Risk	-	Panel of ST2, MMP-3, CXCL9, OPN [11] 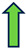
-	-	CXCL9 [9] 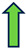
-	-	CD163 [27] 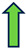

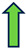
: Increased levels; 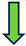: decreased levels.

**Table 2:** miRNA biomarkers in aGVHD and cGVHD.

aGVHD	cGVHD
Genetic Biomarkers	
miR-155 [46,47] 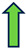	miR17-92 [52,53] 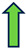
miR181a [46,47] 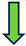	miR-365-3p, miR-148-3p, and miR-378-3p [52,53] (proposed as a panel) 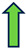
miR20a [49] 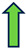	
miR15a [49] 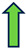	
miR146a [48,49] 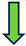	
miR30b-5p [49] 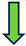	
miR374-5p [49] 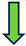	
miR34a-3p and miR503-5p [50] 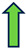	

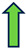
: Upregulation; 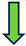: downregulation.

## Abbreviations

IL2-R-a: Interleukin 2 Receptor a; ST2: Suppressor of Tumorigenicity 2; HGF: Hepatocyte Growth Factor; TNFR-1: Tumor Necrosis Factor Receptor-1; Reg-3a: Regenerating Islet-Derived Protein 3a, a peptide found in Paneth cells of the intestines (the most validated GI aGVHD biomarker); TIM 3: T-cell Immunoglobulin Mucin Protein 3; Elafin: a serine protease inhibitor; sBAFF: Soluble B-Cell Activating Factor; CXCL9: Chemokine (C-X-C motif) Ligand 9; CXCL10: Chemokine (C-X-C motif) Ligand 10; DDK3: Dickkopf-Related Protein; MMP-3: Matrix Metalloproteinase 3; MMP-9: Matrix Metalloproteinase; OPN: Osteopontin; CD163: a scavenger receptor by activated monocytes/macrophages found in the case of oxidative stress.
